# Supplementary material for: Enhanced ventilation of Eastern North Atlantic Oxygen Minimum Zone with deglacial slowdown of Meridional Overturning
Source: Nat Commun. 2025 Jul 15;16:6418. doi: 10.1038/s41467-025-61177-3 (PMC12263849; doi:10.1038/s41467-025-61177-3)
Supplement: Supplementary file 1 — Supplementary Information [file 41467_2025_61177_MOESM1_ESM.pdf]

Supplementary Information for *Enhanced ventilation of Eastern North Atlantic Oxygen Minimum Zone with deglacial slowdown of Meridional Overturning*

Sofía Barragán-Montilla[1,2], Heather J. H. Johnstone[1], Stefan Mulitza[1], Dharma A. Reyes Macaya[1,3,4], Babette A. A. Hoogakker[3], Heiko Pälike[1]

[1] MARUM – Center for Marine Environmental Sciences and Department of Geosciences, University of Bremen, Bremen, 28359, Germany

[2] Kiel University, Institute of Geosciences, Kiel, Germany*

[3] Lyell Centre, Heriot-Watt University, Edinburgh, UK

[4] Millennium Nucleus UPWELL, Concepcion, Chile

Correspondence to: Sofía Barragán Montilla (sbarraganmontilla@marum.de)

*Current affiliation

Supplementary Information 1

Benthic Foraminifera Images

Plate 1 – GeoB9512-5

Scale bar 100 µm

1. *Karreriella bradyi* (Cushman, 1911) – 187.5 cm
2. *Sigmoilopsis schlumbergeri* (Silvestri, 1904) – 432.5 cm
3. *Sigmoilopsis schlumbergeri* (Silvestri, 1904) – 452.5 cm
4. *Sigmoilopsis schlumbergeri* (Silvestri, 1904) – 532.5 cm
5. *Reophax* spp. – 262.5 cm
6. *Haplophragmoides* spp. – 262.5 cm
7. *Martinottiella communis* (d'Orbigny, 1846) – 262.5 cm
8. *Martinottiella communis* (d'Orbigny, 1846) – 262.5 cm
9. *Triloculina trigonula* (Lamarck, 1804) – 442.5 cm
10. *Pyrgo williamsoni* (Silvestri, 1923) – 447.5 cm
11. *Pyrgo williamsoni* (Silvestri, 1923) – 417.5 cm
12. *Pyrgo williamsoni* (Silvestri, 1923) – 387.5 cm
13. *Pyrgo comata* (Brady, 1881) – 397.5 cm
14. *Pyrgo comata* (Brady, 1881) – 222.5 cm
15. *Pyrgo depressa* (d'Orbigny, 1826) – 247.5 cm
16. *Pyrgo depressa* (d'Orbigny, 1826) – 252.5 cm
17. *Spiroloculina depressa* d'Orbigny, 1826 – 112.5
18. *Quinqueloculina seminulum* (Linnaeus, 1758) – 227.5 cm
19. *Quinqueloculina seminulum* (Linnaeus, 1758) – 232.5 cm
20. *Quinqueloculina seminulum* (Linnaeus, 1758) – 247.5 cm
21. *Quinqueloculina suborbicularis* d'Orbigny in Fornasini, 1905 – 247.5 cm
22. *Triloculina trigonula* (Lamarck, 1804) – 220 cm
23. *Lagena semistriata* Williamson, 1848 – 267.5 cm
24. *Cushmanina* sp. – 272.5 cm
25. *Lagena semistriata* Williamson, 1848 – 167.5 cm
26. *Lagena* cff. *sulcata* (Walker & Jacob, 1798) – 202.5 cm
27. *Lagena* cff. *sulcata* (Walker & Jacob, 1798) – 152.5 cm
28. *Lagena striata* (d'Orbigny, 1839) – 152.5 cm
29. *Lagena sulcata* (Walker & Jacob, 1798) – 212.5 cm
30. *Cushmanina* sp. – 167.5 cm

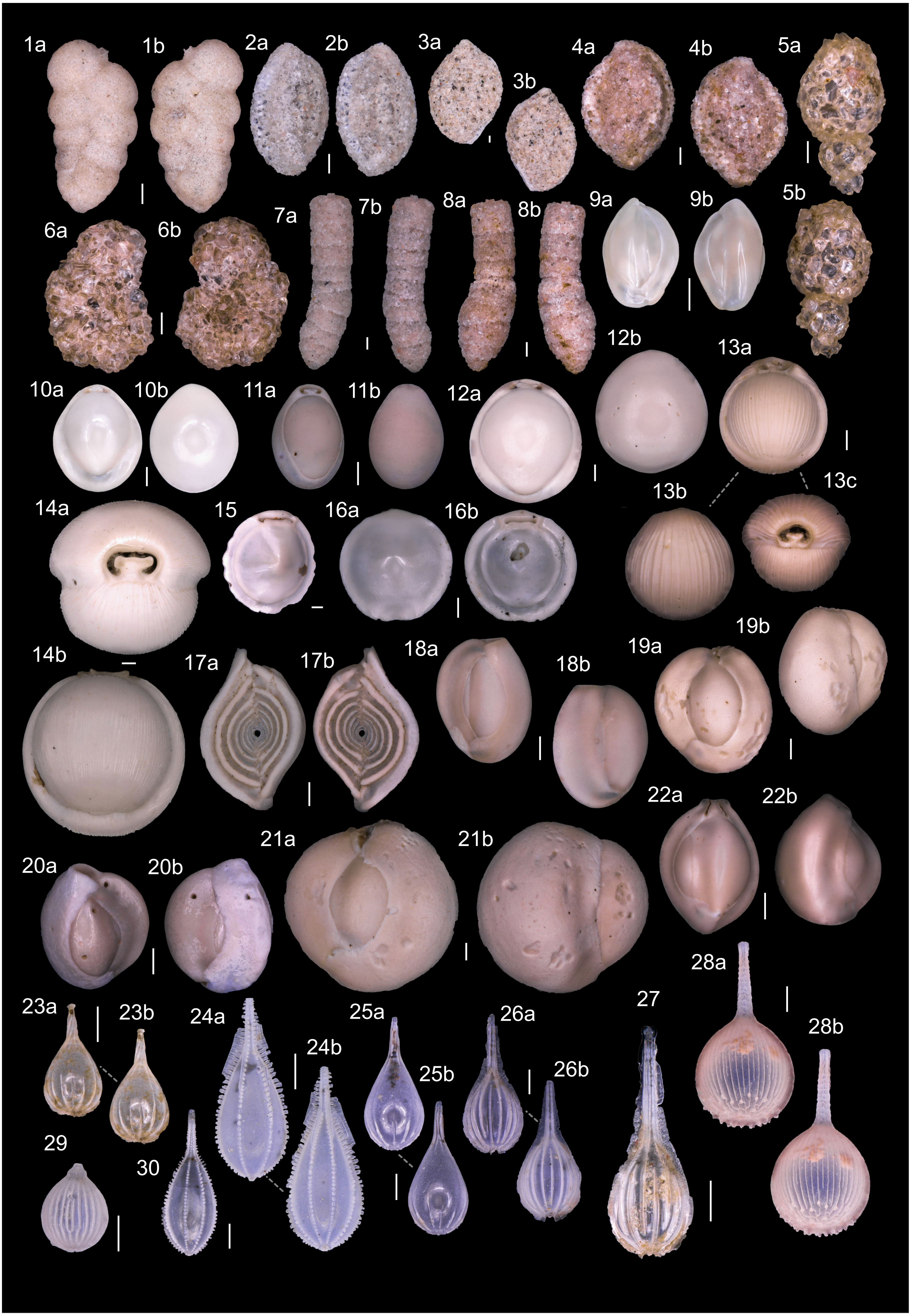
1a
1b
2a
2b
3a
3b
4a
4b
5a
5b
6a
6b
7a
7b
8a
8b
9a
9b
10a
10b
11a
11b
12a
12b
13a
13b
13c
14a
14b
15
16a
16b
17a
17b
18a
18b
19a
19b
20a
20b
21a
21b
22a
22b
23a
23b
24a
24b
25a
25b
26a
26b
27
28a
28b
29
30

Plate 2 – GeoB9512-5

Scale bar 100 µm

1. *Hyalinonetrion gracillimum* (Seguenza, 1862) – 267.5 cm
2. *Grigelis pyrula* (d'Orbigny, 1826) – 262.5 cm
3. *Hyalinonetrion gracillimum* (Seguenza, 1862) – 302.5 cm
4. *Amphicoryna scalaris* (Batsch, 1791) – 312.5 cm
5. *Amphicoryna scalaris* (Batsch, 1791) – 92.5 cm
6. *Uvigerina mediterranea* Hofker, 1932 – 62.5 cm
7. *Uvigerina mediterranea* Hofker, 1932 – 382.5 cm
8. *Uvigerina mediterranea* Hofker, 1932 – 382.5 cm
9. *Bulimina striata* d'Orbigny in Guérin-Méneville, 1832 – 472.5 cm
10. *Uvigerina peregrina* Cushman, 1923 – 2.5 cm
11. *Uvigerina hispida* Schwager, 1866 – 97.5 cm
12. *Bolivina* cff. *spathulata* (Williamson, 1858) – 27.5 cm
13. *Rectuvigerina* cff. *elongatastriata* (Colom, 1952) – 2.5 cm
14. *Bulimina aculeata* d'Orbigny, 1826 – 37.5 cm
15. *Bulimina marginata* d'Orbigny, 1826 – 17.5 cm
16. *Bolivina subaenariensis* var. *mexicana* Cushman, 1922 – 282.5 cm
17. *Bolivina* cff. *spathulata* (Williamson, 1858) – 27.5 cm
18. *Bolivina subaenariensis* var. *mexicana* Cushman, 1922 – 292.5 cm
19. *Bolivina subaenariensis* var. *mexicana* Cushman, 1922 – 277.5 cm
20. *Bolivina* cff. *spathulata* (Williamson, 1858) – 17.5 cm
21. *Globobulimina turgida* (Bailey, 1851) – 72.5 cm
22. *Globobulimina pacifica* Cushman, 1927 – 62.5 cm
23. *Chilostomella oolina* Schwager, 1878 – 82.5 cm
24. *Globobulimina turgida* (Bailey, 1851) – 212.5 cm
25. *Globobulimina turgida* (Bailey, 1851) – 217.5 cm
26. *Globobulimina turgida* (Bailey, 1851) – 257.5 cm
27. *Chilostomella oolina* Schwager, 1878 – 217.5 cm
28. *Glandulina ovula* d'Orbigny, 1846 – 212.5 cm
29. *Fissurina* sp. 1 – 117.5 cm
30. *Fissurina* cff. *staphyllearia* Schwager, 1866 – 117.5 cm
31. *Fissurina* cff. *staphyllearia* Schwager, 1866 – 177.5 cm
32. *Robertinoides bradyi* (Cushman & Parker, 1936) – 437.5 cm
33. *Fursenkoina bradyi* (Cushman, 1922) – 247.5 cm
34. *Fursenkoina bradyi* (Cushman, 1922) – 262.5 cm
35. *Fursenkoina bradyi* (Cushman, 1922) – 262.5 cm
36. *Fursenkoina bradyi* (Cushman, 1922) – 92.5 cm
37. *Nonionella* cff. *pulchella* Hada, 1931 – 102.5 cm

Plate 2

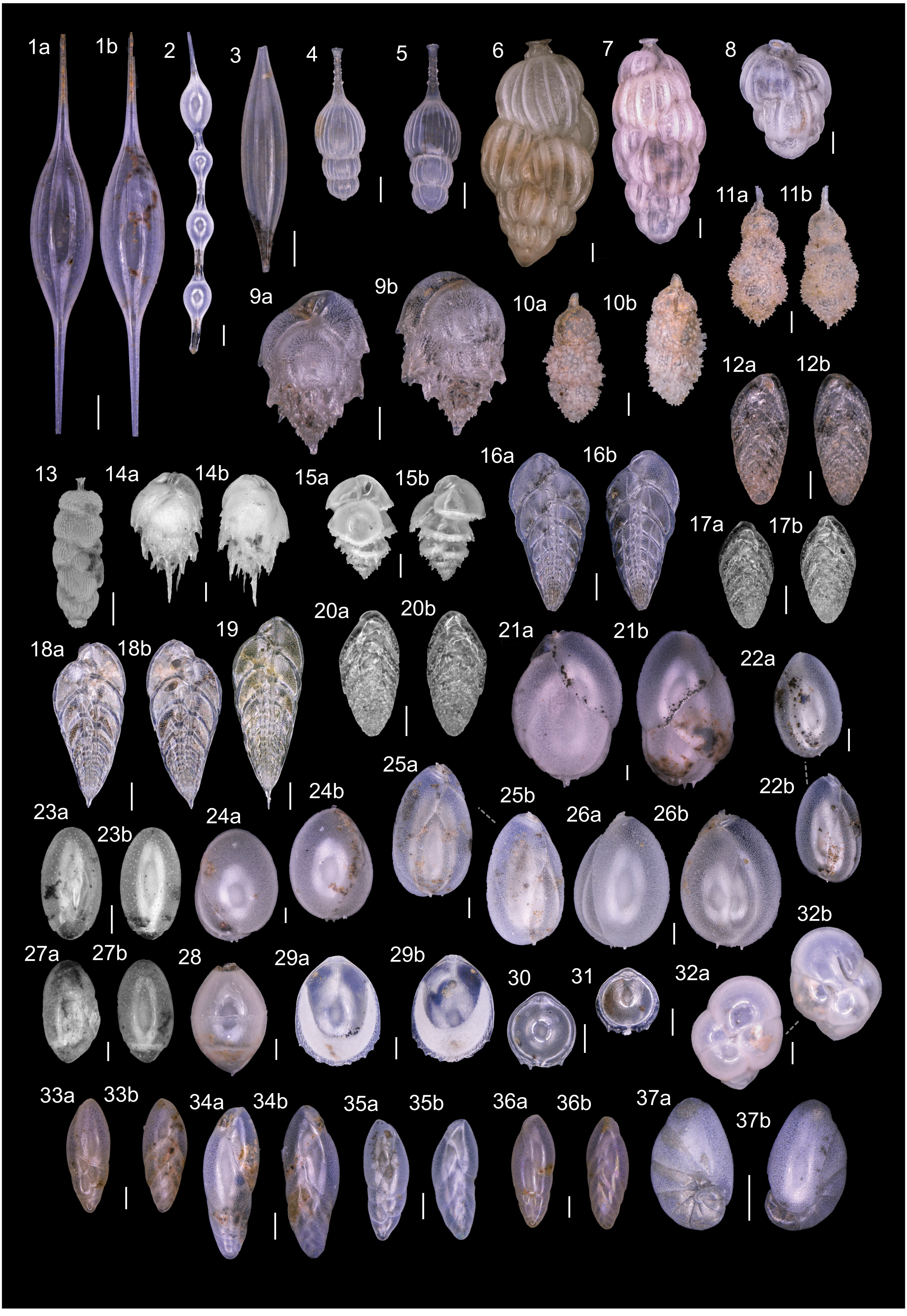

1a
1b
2
3
4
5
6
7
8
9a
9b
10a
10b
11a
11b
12a
12b
13
14a
14b
15a
15b
16a
16b
17a
17b
18a
18b
19
20a
20b
21a
21b
22a
22b
23a
23b
24a
24b
25a
25b
26a
26b
27a
27b
28
29a
29b
30
31
32a
32b
33a
33b
34a
34b
35a
35b
36a
36b
37a
37b

Plate 3 – GeoB9512-5

Scale bar 100 µm

1. *Nonion* cff. *fabum* (Fichtel & Moll, 1798) – 102.5 cm
2. *Nonion* cff. *fabum* (Fichtel & Moll, 1798) – 192.5 cm
3. *Nonionella* cff. *pulchella* Hada, 1931 – 187.5 cm
4. *Cancris auriculus* (Fichtel & Moll, 1798) – 447.5 cm
5. *Cancris auriculus* (Fichtel & Moll, 1798) – 462.5 cm
6. *Neolenticulina variabilis* (Reuss, 1850) – 242.5 cm
7. *Nonion* cff. *fabum* (Fichtel & Moll, 1798) – 207.5 cm
8. *Cancris auriculus* (Fichtel & Moll, 1798) – 57.5 cm
9. *Sphaeroidina bulloides* d'Orbigny in Deshayes, 1832 – 22.5 cm
10. *Sphaeroidina bulloides* d'Orbigny in Deshayes, 1832 – 42.5 cm
11. *Neolenticulina variabilis* (Reuss, 1850) – 212.5 cm
12. *Cassidulina laevigata* d'Orbigny, 1826 – 27.5 cm
13. *Oridorsalis umbonatus* (Reuss, 1851) – 127.5 cm
14. *Oridorsalis umbonatus* (Reuss, 1851) – 147.5 cm
15. *Cassidulina laevigata* d'Orbigny, 1826 – 27.5 cm
16. *Hanzawaia boueana* (d'Orbigny, 1846) – 97.5 cm
17. *Hanzawaia boueana* (d'Orbigny, 1846) – 132.5 cm
18. *Cibicidoides wuellerstorfi* (Schwager, 1866) – 87.5 cm
19. *Planulina ariminensis* d'Orbigny, 1826 - 92.5 cm
20. *Planulina ariminensis* d'Orbigny, 1826 - 72.5 cm
21. *Cibicidoides robertsonianus* (Brady, 1881) – 102.5 cm
22. *Lenticulina iota* (Cushman, 1923) – 232.5 cm
23. *Hyalinea balthica* (Schröter, 1783) – 482.5 cm
24. *Melonis barleeanus* (Williamson, 1858) – 457.5 cm
25. *Pullenia bulloides* (d'Orbigny, 1846) – 132.5 cm

Plate 3

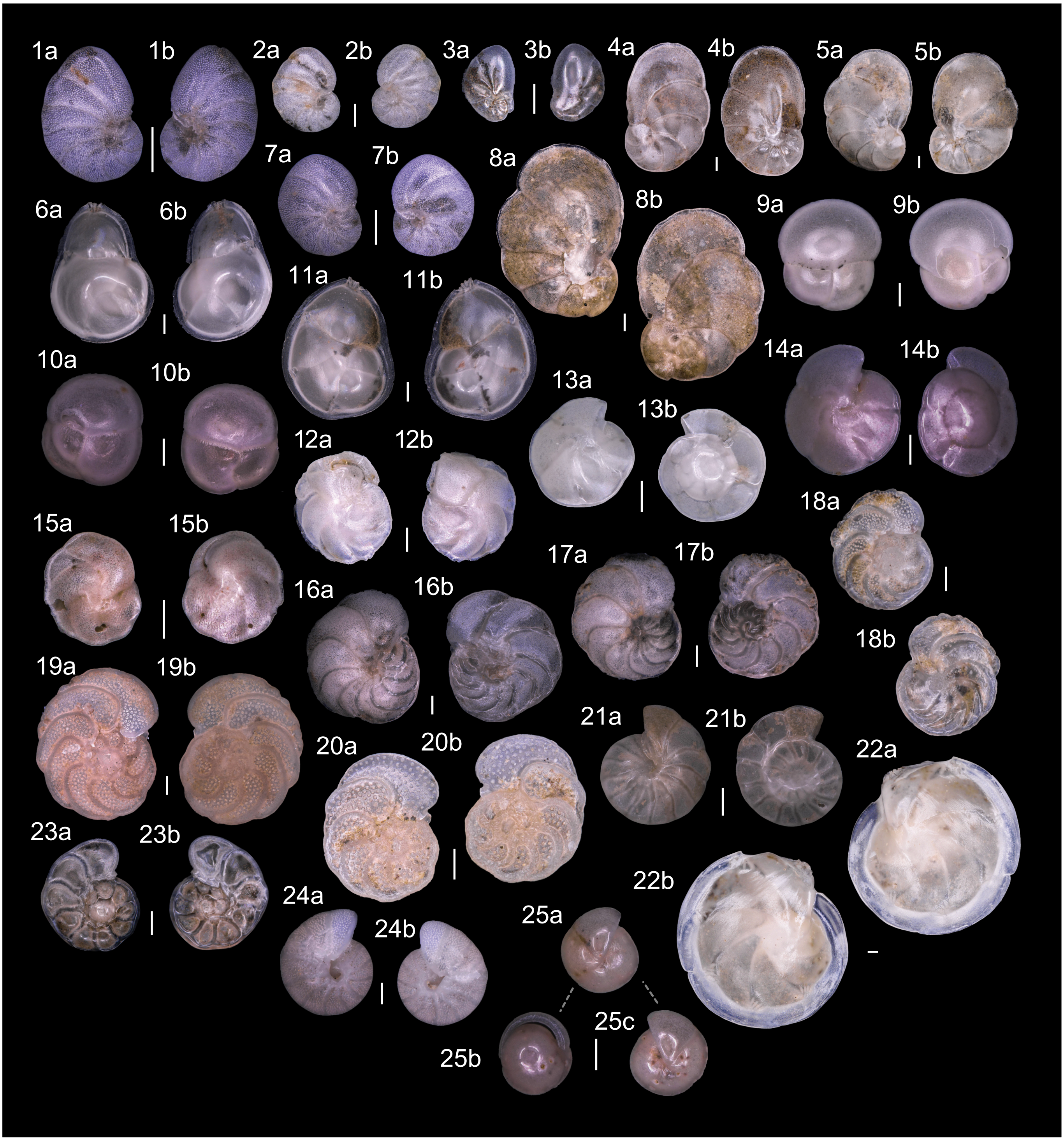

1a
1b
2a
2b
3a
3b
4a
4b
5a
5b
6a
6b
7a
7b
8a
8b
9a
9b
10a
10b
11a
11b
12a
12b
13a
13b
14a
14b
15a
15b
16a
16b
17a
17b
18a
18b
19a
19b
20a
20b
21a
21b
22a
22b
23a
23b
24a
24b
25a
25b
25c

Supplementary Information 2 - Enhanced Benthic Foraminifera Index application in the Tropical Eastern Atlantic: improved benthic foraminifera oxygen preferences database and alternative calibration

The Enhanced Benthic Foraminifera Oxygen Index[1] (EBFOI) presents an improvement of the BFOI[2], which is used to reconstruct paleo-oxygenation changes using benthic foraminifera taxonomy. This improvement included the addition of more agglutinated species, as well as the addition of samples from different ocean basins, providing a wider geographical database of the oxygen preferences of benthic foraminifera species[1]. With this approach, the EBFOI provides a more accurate approach to oxygen reconstructions, and allows for a more reliable interpretation of the benthic foraminifera record for areas like the Eastern Tropical North Atlantic Oxygen Minimum Zone (ETNA OMZ) where oxygen is depleted (to values of < 40 µmol/kg in its core[3], but is not as low as, for instance, the Eastern Tropical South Atlantic Oxygen Minimum Zone (approximately < 20 µmol/kg at its core[3]).

As with any other paleoceanographic proxy, the EBFOI[1] still has some limitations[4] as incomplete information about oxygen preferences of benthic foraminifera species can result in an underestimation of oxygen concentrations, especially in certain regions. In order to improve the proxy, the EBFOI was furthered refined by adding more information about additional Atlantic species[4].

For our oxygen reconstruction presented in this study, we used the original calibration

and transfer function[1], but we revised the available information about the oxygen preferences of the benthic foraminifera species in the area, to produce an updated oxygen preference compilation (Supplementary Data 3). We combined published oxygen preference data[1,2,4] (Supplementary Data 3, Sheet 6), which gave data on over 900 species. In order to provide more information about the east tropical Atlantic we then added data published for living assemblages of benthic foraminifera from box and sediment cores from the eastern tropical Atlantic (Figure S2.1; Supplementary Data 4)[5–7].

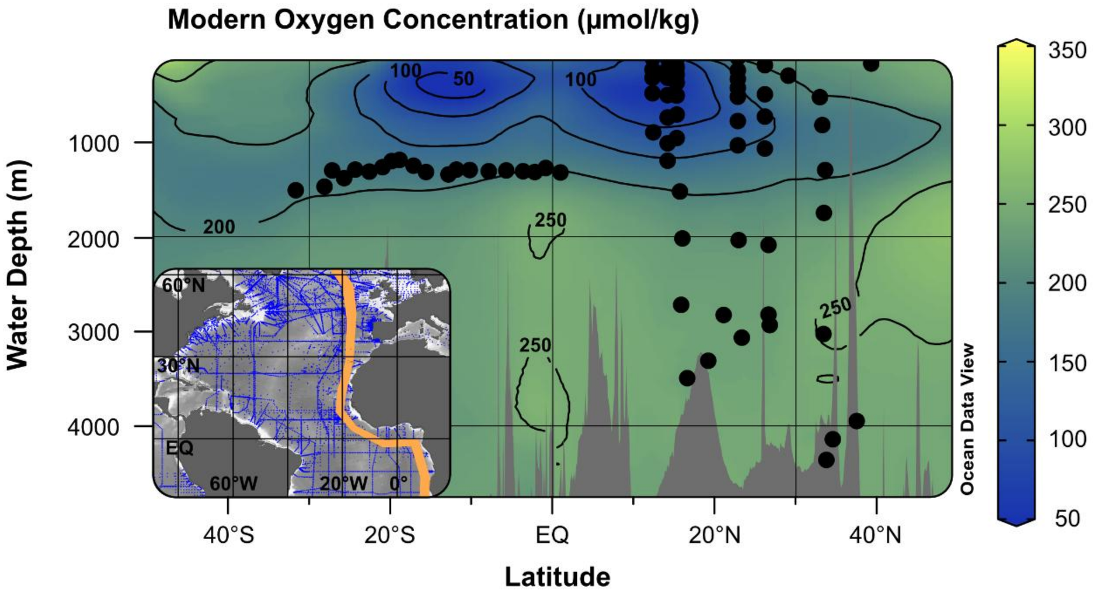

Figure S2.1 Sites of living foraminifera data used in this study. Black markers indicate sample sites[5–7]. Oxygen data extracted from GLODAP version 2.2022[8]; Bathymetry information as reported in the datasets at PANGAEA. Plotted using Ocean Data View[9]. Inset shows the selected GLODAP section referred to in the text.

This data consists of percentages of living benthic foraminifera from 141 sites (Figure S2.1) where around 216 species are reported (Supplementary Data 4). Oxygen concentrations were not given in the original publications. To obtain oxygen values of

bottom water for all the sites in our new compilation, we extracted data from the curated hydrological data set GLODAP (The Global Ocean Data Analysis Project, https://glodap.info/) version 2.2022[8]. Specific oceanic sections were selected (40ºN - 40ºS; along the western European and African continental margin). The selected section is shown in Figure S2.1 (inset). Temperature in-situ (ºC), salinity, oxygen (µmol/kg), pH, alkalinity (µmol/kg) and $\delta^{13}C$ of dissolved inorganic carbon ($\delta^{13}C_{DIC}$) (‰) original curated GLODAP v2.2022 data set[8], were interpolated using Data Interpolated Variational Analysis (referred to as DIVA-interpolation) and Weighted Average Interpolation without outliers of Ocean Data View with maximum gridding of 35*X**25*Y* with a quality limit of 3.0 and excluding outliers. The Python script Bottom Water Calculations [Python code is available at https://github.com/Paleobiogeochemistry/BOT] was used to find the closest interpolated datapoint to the latitude/longitude/water depth of each sample.

Once we had oxygen data for all the sites, we found the maximum and minimum oxygen values where each species was abundant (relative abundance >10%). Then, to establish with a degree of certainty if a species was oxic (>65.4 µmol/kg (>3ml/l)), suboxic (13.5 - 65.4 µmol/kg (0.3 – 3 ml/l)) or dysoxic (<13.5 µmol/kg (<0.3 ml/l)) we only considered species which were abundant more than 1 of the 141 sites. This methodology allowed us to assign oxygen categories to 91 species (Supplementary Data 3, Sheet 6; Supplementary Data 4, Sheet 4). We note that the compilation is dominated by oxygenated environments, as only 10 sites are located in suboxic oxygen conditions below 65.4 µmol/kg and no sites are reported in dysoxic conditions.

We then compared our eastern tropical Atlantic living assemblage data with compiled data[1,2,4]. If the assigned oxygen category for a species in our living assemblage data was in agreement with at least two out of the three cited data sets, then it was retained in our final compilation (total compile of this study in Supplementary Data 3, sheet 6).

In order to test our new benthic oxygen preference compilation, we counted benthic forams in 26 core-tops (dead assemblages) from the eastern tropical Atlantic[10–12]. Counts are given in Supplementary Data 3. Eleven of these samples proved to either have too few benthic foraminifera, or too little information (<80%) about the oxygen preferences of species present, to provide meaningful assemblage data. For the other 15 core-top samples we calculated the EBFOI. Modern oxygen concentrations for the sites were obtained using the python script, BOT, as described above. There was a strong correlation (R^2 =0.8) between EBFOI and modern oxygen concentration (Figure S2.2) confirming the reliability of this proxy in our area.

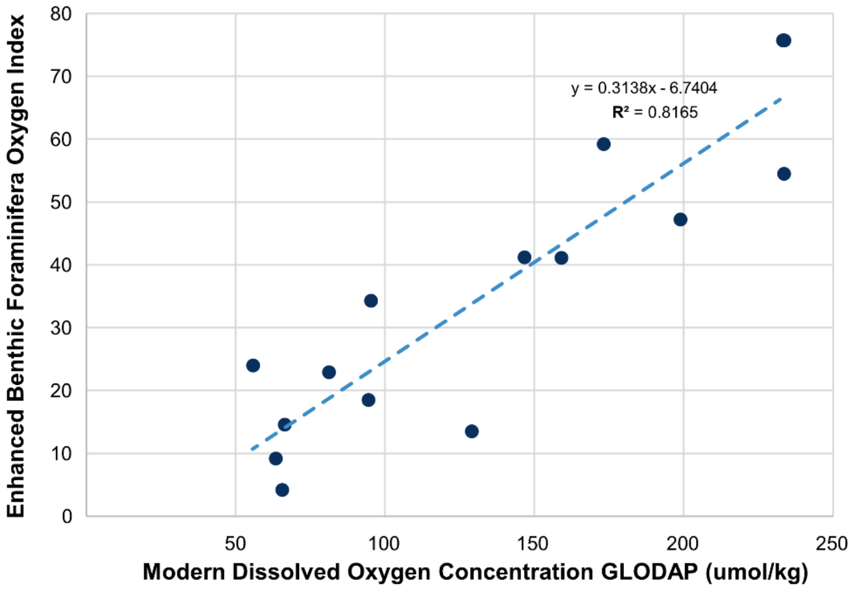

Figure S2.2. Relationship between Bottom and Pore Water Oxygen (BPWO, umol/kg) and Enhanced Benthic Foraminifera Oxygen Index (EBFOI) calculated for 15 core-tops from the tropical Atlantic using the oxygen preference compilation of this study. Oxygen values for the core-top sites were estimated using GLODAP (The Global Ocean Data Analysis Project, https://glodap.info/) version 2.2022[8].

Additionally, to evaluate the accuracy of the different benthic foraminifera oxygen preference compilations we calculated the oxygen concentration from EBFOI of the 15 core-top samples using the calibration equation of Kranner et al. (2022) and three different oxygen preference compilations - that of Kranner et al., (2022), that of Schmiedl (2023) and the compilation from this study ("total compile"). We then calculated the difference between PBWO obtained from EBFOI and the modern bottom water oxygen concentration (Figure S2.3). We find that oxygen values calculated using the compilation from this study and that of Schmiedl et al. (2023) approximate more closely to modern values than those using exclusively the oxygen preference compilation of Kranner et al., (2022).

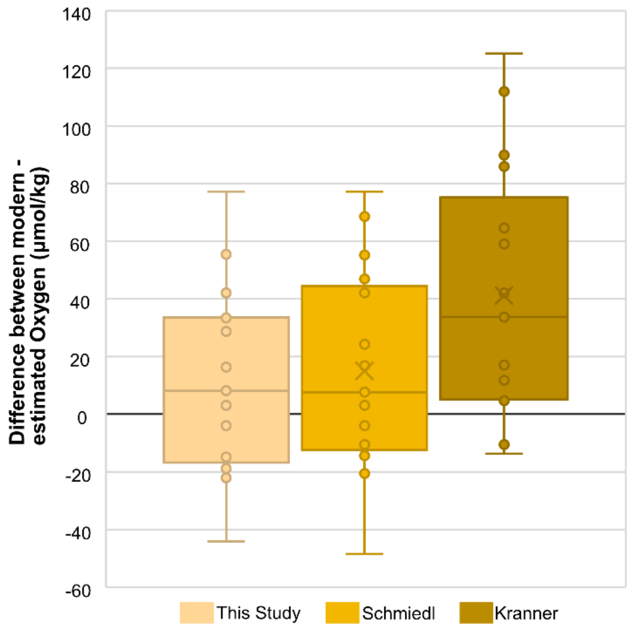

Figure S2.3 Box plot showing differences between Bottom and Pore Water Oxygen (BPWO) calculated from benthic foraminifera and the modern bottom water oxygen (from GLODAP v2.2022[8]) for 15 core- tops from the Atlantic[12] using three different oxygen preference compilations. The symbol (x) represents the mean.

A similar comparison, using four different oxygen preference compilations, for GeoB9512 data (Fig S4.4) also shows differences in BPWO reconstructed using the different compilations. The record using Kranner et al. (2022) benthic foraminifera oxygen preferences results in generally lower oxygen values, and no increase in oxygen in the latter part of the Heinrich Stadial 1 (HS1) (orange curve in Figure S2.4), although three out of four compilations do suggest increased oxygen at this time. Three of the four compilations suggest a rise in oxygen at ~16 ka, although using the oxygen preference data of Schmiedl et al. (2023) alone (black line in S4.4) does not indicate increased BPWO at this time. We find that oxygen underestimation (one of the major limitations of Kranner et al. (2022) EBFOI), is substantially decreased by adding more benthic foraminifera oxygen preferences for Atlantic species.

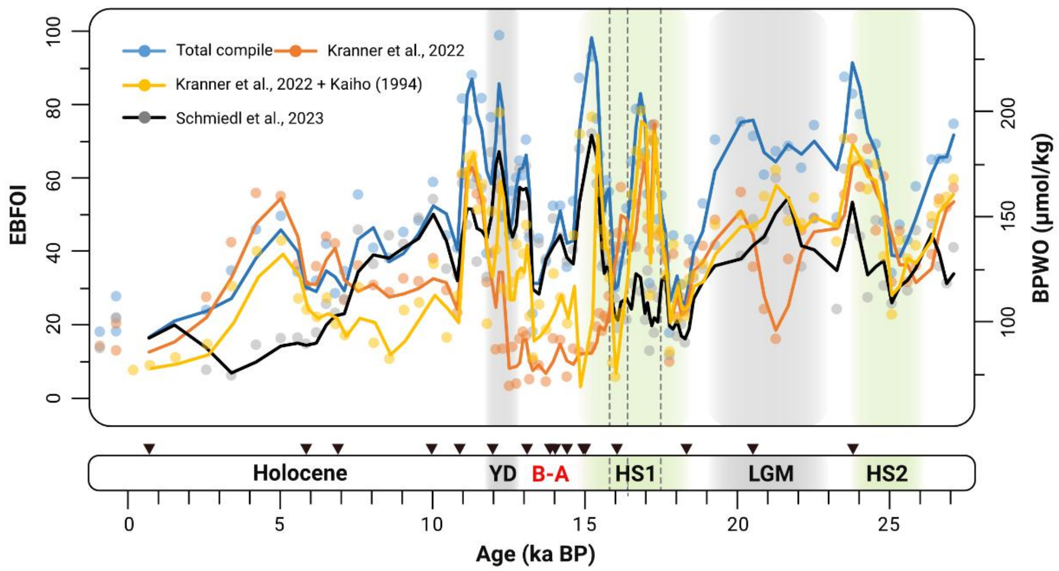

Figure S2.4 GeoB95812-5 Enhanced Benthic Foraminifera Oxygen Index (dots, EBFOI) and 2-point moving average Bottom and Pore Water Oxygen (curves, BPWO - µmol/kg) calculated using the calibration equation of Kranner et al., (2022) and four different benthic foraminifera oxygen preference compilations.

In this study, we interpret the oxygen record of GeoB9512-5 based on the EBFOI calculated using our updated benthic foraminifera oxygen preference database (blue curve in Figure S2.4, Supplementary Data 4, sheet 1), and the transfer function of Kranner et al. (2022).

Supplementary Information 3 - Relation to other deglacial paleo-oxygenation records

The intermediate Atlantic was oxygenated in times of deep AMOC slowdown as seen by the global contrast between LGM and Holocene Ocean paleo-oxygen concentrations across different ocean basins (Figure S3.1b)[13]. The authors found a lower-than-Holocene LGM global oxygenation, however, a considerable number of the cited records show higher ocean oxygenation during the LGM compared to the Holocene, especially from records shallower than 1.500 m water depth[13].

In fact, some evidence shows less oxygen in the deep North Atlantic during the LGM compared to the Holocene[14]. According to the study, this oxygen concentration decrease is related to higher respired carbon storage due to a replacement of northern-sourced deep waters by southern-sourced waters in the deep Atlantic, an observation supported by other records of $\delta^{13}C$ and CdW in the Atlantic[14]. On the other hand, a consensus on whether the ocean oxygenation of the upper North Atlantic was higher or lower during

the LGM remains elusive due to the limited records, especially from lower latitudes[14].

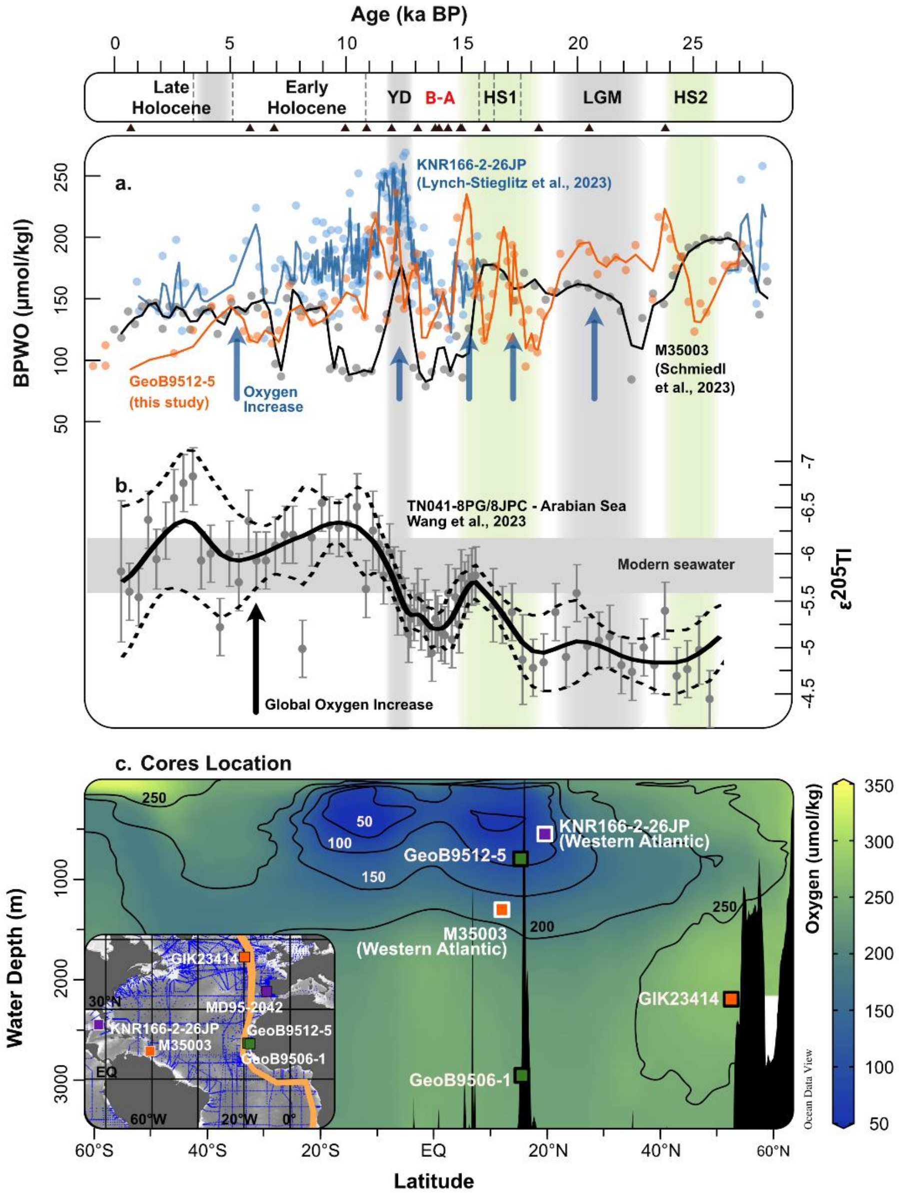

Figure S3.1. Deglacial Benthic and Pore Water Oxygen (BPWO, µmol/kg) records from the western and eastern upper tropical Atlantic. BPWO records from the last 30.000 years in the **a.** upper tropical Atlantic (GeoB9512-5 this study); KNR166-2-26JP (24°19.61′ N, 83°15.14′ W, 546 m water depth)[15]; and M35003 (12°05.1´N, 61°14.1´W, 1.299 m water depth)[4]; and **b.** Global Oxygenation Inferred from site

TN041-8PG/8JPC in the Arabian Sea, dashed curve indicates the bootstrapped 2-SD envelope, and error bars are 2 SD (17°48.76′N, 57°30.34′E, 761 m water depth)[13]; **c.** Location of other high- resolution paleo-oxygenation records in the tropical North Atlantic used here. Oxygen data is from GLODAP v2.2022 oxygen data base[8], plotted with Ocean DataView[9]. Inset shows the selected GLODAP sections referred to in the text. **Key Climate events:** Heinrich Stadial 2 (HS2); Last Glacial Maximum (LGM); Heinrich Stadial 1 (HS1); Bølling–Allerød (B-A); Younger Dryas (YD). Triangles in age axis indicate radiocarbon ages of site GeoB9512-5.

A recent oxygen reconstruction based on benthic foraminifera carbon isotopic offset $\Delta\delta^{13}C$ (between *Cibicidoides* and *Globobulimina)* showed that at least for the YD in the western tropical Atlantic (blue line in Figure S3.1a, KNR166-2-26JP, 24°19.61′ N, 83°15.14′ W, 546 m water depth), intermediate waters became notably more oxygenated[15]. This is consistent with our observations (green line in Figure S3.1a) and those of the EBFOI-based M35003 paleo-oxygenation record (black line in Figure S3.1a, 12°05.1´N, 61°14.1´W retrieved at 1.299 m) southeast of the Island of Grenada in the Caribbean[4]. The latter study contrasted the benthic foraminifera diversity and calculated the dissolved oxygen concentration using the EBFOI of different records across ocean basins[4]. The authors indeed observed an average increase of oxygen in the North Atlantic during the HS1 and YD, alternating with a marked drop during the B-A.

Oxygenated intermediate eastern tropical Atlantic in times of enhanced ocean productivity and ocean warming

Other factors influencing oxygen concentration in modern oceans are temperature and nutrient content. Higher temperatures decrease oxygen solubility, limiting its

concentration in ocean waters[16,17]. On the other hand, organic matter and nutrient accumulation lead to oxygen consumption from organisms[18], also decreasing oxygen concentrations. Limited nutrient supply due to the reduced presence of AAIW during AMOC slowdown in the YD is evidenced by the oxygen increase in the intermediate western tropical Atlantic[15]. These results are supported by decreasing TOC (wt %) in the YD and HS1 at site M35003-4[19], which would explain the higher oxygen during the HS1 and YD in the same site (M35003[4]). However, such an explanation cannot be transferred to GeoB9512-5. Oxygenated periods in the GeoB9512-5 record during HS1 and the YD coincide with increased productivity recorded at several sites along the West African coast[20] (Figure 2c-d in Manuscript[21–23]), attributed to enhanced upwelling related to stronger NE trade winds. High productivity suggests adequate nutrient content during these times in the tropical NE Atlantic. The infaunal foraminifera content at our site is always fairly high (on average > 62%) and indicates meso-eutrophic conditions (high organic matter concentrations) at the seafloor throughout the record.

Available records from the western Atlantic[24–27] and eastern Atlantic[28], show intense warming episodes above 1.500 m in the tropical Atlantic during the HS1 and YD, also related to a heat accumulation in the shallower ocean in times of reduced AMOC circulation. Strikingly, such warming did not lead to a decline in oxygenation for the western and eastern tropical Atlantic. Climate model simulations show that in times of reduced Atlantic thermohaline circulation, the equatorial flow of the Subtropical Cell strengthens and acts like a closed cell that transports warmer and saltier waters into the equatorial zone[29]. This explains the intense warming in the mid-depth eastern[28] and

western Atlantic[24–27] and the synchronous higher oxygenation observed in our study and the western tropical Atlantic[4,15], which therefore would be consistent with higher subsurface renewal rates from a stronger subtropical gyre circulation.

References

1. Kranner, M., Harzhauser, M., Beer, C., Auer, G. & Piller, W. E. Calculating dissolved marine oxygen values based on an enhanced Benthic Foraminifera Oxygen Index. *Sci. Rep.* **12**, 1376 (2022).
2. Kaiho, K. Benthic foraminiferal dissolved-oxygen index and dissolved-oxygen levels in the modern ocean. *Geology* **22**, 719 (1994).
3. Karstensen, J., Stramma, L. & Visbeck, M. Oxygen minimum zones in the eastern tropical Atlantic and Pacific oceans. *Prog. Oceanogr.* **77**, 331–350 (2008).
4. Schmiedl, G., Milker, Y. & Mackensen, A. Climate forcing of regional deep-sea biodiversity documented by benthic foraminifera. *Earth-Sci. Rev.* **244**, 104540 (2023).
5. Haake, F. W. Benthic foraminifera of surface samples and sediment cores off Senegal and Gambia, West Africa. 4 datasets Preprint at https://doi.org/10.1594/PANGAEA.548467 (1980).
6. Licari, L. & Mackensen, A. Assemblage of living benthic foraminifera in sediment core GeoB3715-1. 1304 data points PANGAEA https://doi.org/10.1594/PANGAEA.511360 (2006).
7. Lutze, G. F. Distribution of benthic foraminifera in surface sediments on the continental margin off North-West Africa. 6 datasets Preprint at https://doi.org/10.1594/PANGAEA.548469 (1980).

8. Lauvset, S. K. *et al.* GLODAPv2.2022: the latest version of the global interior ocean biogeochemical data product. *Earth Syst. Sci. Data* **14**, 5543–5572 (2022).
9. Schlitzer, R. Ocean Data View, , odv.awi.de. (2023).
10. Mulitza, S. *et al.* Report and preliminary results of METEOR Cruise M65/1, Dakar - Dakar, 11.06. - 1.07.2005. (2005).
11. Jansen, J. H. F., De Lange, G. J. & van Bennekom, A. J. (Pale)oceanography and geochemistry of the Angola Basin (South Atlantic Ocean) : cruise report R.V. Tyro 30 September-19 November. 11–65 (1990).
12. Barragán-Montilla, S. Benthic Foraminifera counts off NW Africa during the last deglaciation. 45030 data points PANGAEA https://doi.org/10.1594/PANGAEA.962951 (2024).
13. Wang, Y., Costa, K. M., Lu, W., Hines, S. K. V. & Nielsen, S. G. Global oceanic oxygenation controlled by the Southern Ocean through the last deglaciation. *Sci. Adv.* **10**, eadk2506 (2024).
14. Zhou, Y. & McManus, J. F. Authigenic uranium deposition in the glacial North Atlantic: Implications for changes in oxygenation, carbon storage, and deep water-mass geometry. *Quat. Sci. Rev.* **300**, 107914 (2023).
15. Lynch-Stieglitz, J. *et al.* A diminished North Atlantic nutrient stream during Younger Dryas climate reversal. *Science* **384**, 693–696 (2024).
16. Schmidtko, S., Stramma, L. & Visbeck, M. Decline in global oceanic oxygen content during the past five decades. *Nature* **542**, 335–339 (2017).
17. Gilbert, D. Oceans lose oxygen. *Nature* **542**, 303–304 (2017).
18. Arndt, S. *et al.* Quantifying the degradation of organic matter in marine sediments: A

review and synthesis. *Earth-Sci. Rev.* **123**, 53–86 (2013).

19. Vink, A. *et al.* Shifts in the position of the north equatorial current and rapid productivity changes in the western tropical Atlantic during the last glacial. *Paleoceanography* **16**, 479–490 (2001).
20. Zarriess, M. & Mackensen, A. The tropical rainbelt and productivity changes off northwest Africa: A 31,000-year high-resolution record. *Mar. Micropaleontol.* **76**, 76–91 (2010).
21. Bouimetarhan, I., Groeneveld, J., Dupont, L. & Zonneveld, K. Low- to high-productivity pattern within Heinrich Stadial 1: Inferences from dinoflagellate cyst records off Senegal. *Glob. Planet. Change* **106**, 64–76 (2013).
22. Bradtmiller, L. I. *et al.* Changes in biological productivity along the northwest African margin over the past 20,000 years: AFRICAN MARGIN PALEOPRODUCTIVITY. *Paleoceanography* **31**, 185–202 (2016).
23. Romero, O. E., Kim, J. & Donner, B. Submillennial-to-millennial variability of diatom production off Mauritania, NW Africa, during the last glacial cycle. *Paleoceanography* **23**, 2008PA001601 (2008).
24. Came, R. E. *et al.* North Atlantic intermediate depth variability during the Younger Dryas: Evidence from benthic foraminiferal Mg/Ca and the GFDL R30 Coupled Climate Model. in *Geophysical Monograph Series* (eds. Schmittner, A., Chiang, J. C. H. & Hemming, S. R.) vol. 173 247–263 (American Geophysical Union, Washington, D. C., 2007).
25. Oppo, D. W. *et al.* Deglacial Temperature and Carbonate Saturation State Variability in the Tropical Atlantic at Antarctic Intermediate Water Depths. *Paleoceanogr.*

Paleoclimatology **38**, e2023PA004674 (2023).

26. Poggemann, D. -W. *et al.* Deglacial Heat Uptake by the Southern Ocean and Rapid Northward Redistribution Via Antarctic Intermediate Water. *Paleoceanogr. Paleoclimatology* **33**, 1292–1305 (2018).
27. Rühlemann, C. *et al.* Intermediate depth warming in the tropical Atlantic related to weakened thermohaline circulation: Combining paleoclimate data and modeling results for the last deglaciation. *Paleoceanography* **19**, 2003PA000948 (2004).
28. Weldeab, S., Friedrich, T., Timmermann, A. & Schneider, R. R. Strong middepth warming and weak radiocarbon imprints in the equatorial Atlantic during Heinrich 1 and Younger Dryas. *Paleoceanography* **31**, 1070–1082 (2016).
29. Chang, P. *et al.* Oceanic link between abrupt changes in the North Atlantic Ocean and the African monsoon. *Nat. Geosci.* **1**, 444–448 (2008).
