# Supplementary material for: Enhanced ventilation of Eastern North Atlantic Oxygen Minimum Zone with deglacial slowdown of Meridional Overturning
Source: Nat Commun. 2025 Jul 15;16:6418. doi: 10.1038/s41467-025-61177-3 (PMC12263849; doi:10.1038/s41467-025-61177-3)
Supplement: Supplementary file 2 — Description of Additional Supplementary Files [file 41467_2025_61177_MOESM2_ESM.pdf]

- **Supplementary Information 1:** Benthic Foraminifera Images
- **Supplementary Information 2:** Enhanced Benthic Foraminifera Index application in the Tropical Eastern Atlantic: improved benthic foraminifera oxygen preferences database and alternative calibration
- **Supplementary Information 3:** Relation to other deglacial paleo-oxygenation records

- **Supplementary Data 1:** Paleoenvironmental parameters from site GeoB9512-5 estimated per sample
- **Supplementary Data 2:** Benthic Foraminifera counts and information from samples of gravity core GeoB9512-5 and GeoB9512-4 (Multicorer 2-3cm sediment depth), GeoB9512-4 (Multicorer 8-9 cm sediment depth)
- **Supplementary Data 3:** Revised Benthic Foraminifera oxygen Preferences.
  - **Sheet 1:** Counts of benthic foraminifera species from GeoB9512-5, GeoB9806-1 and eastern Atlantic coretops, and EBFOI – Bottom and Pore Water Oxygen estimations (https://doi.pangaea.de/10.1594/PANGAEA.962951)
  - **Sheet 2:** Relative abundances (%) of benthic foraminifera species from GeoB9512-5, GeoB9506-1 and eastern Atlantic coretops
  - **Sheet 3:** Comparison of EBFOI and Bottom and Pore Water Oxygen using different benthic foraminifera databases
  - **Sheet 4:** Linear regression of estimated EBFOI and modern dissolved oxygen concentrations form 15 coretops of the eastern Atlantic
  - **Sheet 5:** Modern vs. Estimated dissolved oxygen concentrations using different benthic foraminifera databases in the calibration of Kranner et al. (2022)
  - **Sheet 6:** Data used for this study benthic foraminifera oxygen preferences compilation
- **Supplementary Data 4:** Living Benthic Foraminifera of the Eastern North Atlantic.
  - **Sheet 1:** Sites and Hidrological Information
  - **Sheet 2:** Benthic Foraminifera species and Counts
  - **Sheet 3:** 3-Suboxic Sites Information
  - **Sheet 4:** 3-Oxic Sites Information
  - **Sheet 5:** Summary
